# When birds of a feather flock together: Severe genomic erosion and the implications for genetic rescue in an endangered island passerine

**DOI:** 10.1111/eva.13739

**Published:** 2024-06-28

**Authors:** Emily L. Cavill, Hernán E. Morales, Xin Sun, Michael V. Westbury, Cock van Oosterhout, Wilna Accouche, Anna Zora, Melissa J. Schulze, Nirmal Shah, Pierre‐André Adam, M. de L. Brooke, Paul Sweet, Shyam Gopalakrishnan, M. Thomas P. Gilbert

**Affiliations:** ^1^ The Globe Institute, University of Copenhagen Copenhagen Denmark; ^2^ School of Environmental Sciences University of East Anglia, Norwich Research Park Norwich UK; ^3^ Green Islands Foundation Victoria Seychelles; ^4^ Fregate Island Sanctuary Ltd Victoria Seychelles; ^5^ Cousine Island Company Providence Seychelles; ^6^ Nature Seychelles Roche Caiman Seychelles; ^7^ Island Conservation Society Pointe Larue Seychelles; ^8^ Department of Zoology University of Cambridge Cambridge UK; ^9^ American Museum of Natural History New York USA; ^10^ University Museum, Norwegian University of Science and Technology Trondheim Norway

**Keywords:** avian conservation, endangered species, genetic bottleneck, genetic rescue, genomic erosion, museomics

## Abstract

The Seychelles magpie‐robin's (SMR) five island populations exhibit some of the lowest recorded levels of genetic diversity among endangered birds, and high levels of inbreeding. These populations collapsed during the 20th century, and the species was listed as Critically Endangered in the IUCN Red List in 1994. An assisted translocation‐for‐recovery program initiated in the 1990s increased the number of mature individuals, resulting in its downlisting to Endangered in 2005. Here, we explore the temporal genomic erosion of the SMR based on a dataset of 201 re‐sequenced whole genomes that span the past ~150 years. Our sample set includes individuals that predate the bottleneck by up to 100 years, as well as individuals from contemporary populations established during the species recovery program. Despite the SMR's recent demographic recovery, our data reveal a marked increase in both the genetic load and realized load in the extant populations when compared to the historical samples. Conservation management may have reduced the intensity of selection by increasing juvenile survival and relaxing intraspecific competition between individuals, resulting in the accumulation of loss‐of‐function mutations (i.e. severely deleterious variants) in the rapidly recovering population. In addition, we found a 3‐fold decrease in genetic diversity between temporal samples. While the low genetic diversity in modern populations may limit the species' adaptability to future environmental changes, future conservation efforts (including IUCN assessments) may also need to assess the threats posed by their high genetic load. Our computer simulations highlight the value of translocations for genetic rescue and show how this could halt genomic erosion in threatened species such as the SMR.

## 1 INTRODUCTION

We are facing a biodiversity crisis in which species are being lost at a faster rate than at any other time in human history (Ceballos et al., 2015). The Seychelles archipelago is located within one of Earth's 36 biodiversity hotspots (Myers, 1988; Noss et al., 2015), that is, areas designated as conservation priorities. Nevertheless, over the past 150 years, the Seychelles has suffered six known avian extinctions (Cheke, 2013; Hume & Walters, 2012), and more than half of the surviving endemic bird species face some level of threat of extinction (IUCN, 2023).

Extinction risk is heightened when species exist in small, fragmented populations (Crooks et al., 2017). Thus, the International Union for Conservation of Nature (IUCN) utilizes population size as one of the key criteria for classifying species as Endangered (IUCN Standards and Petitions Committee, 2022). Anthropogenic actions that can affect the range and population size of species include poaching, the introduction of alien species that outcompete native species, the removal of native habitat, introduction of disease, pollution of land and sea and climate change (Barnosky et al., 2011). These threats can lead to population contractions, which increases genetic drift and leads to genomic erosion. Populations affected by genomic erosion are characterized by diminished adaptive potential, increased genetic load (an accumulation of deleterious alleles) and maladaptation of genetic variants to the environment (e.g. Bosse & van Loon, 2022; van Oosterhout et al., 2022), ultimately hindering both evolutionary potential and long‐term viability. Furthermore, population bottlenecks can increase levels of inbreeding, which increases homozygosity of recessive deleterious mutations (Bertorelle et al., 2022). Before the bottleneck, most of these mutations would have been heterozygous and present as a masked load, which does not impair fitness. However, when mutations become homozygous, the masked load is converted into a realized load, which results in inbreeding depression (Bertorelle et al., 2022). While some of the realized genetic load may eventually be eliminated by purifying selection, the purging of mutations reduces individual fitness and population viability (Crow, 1958; Dussex et al., 2023; Keller & Waller, 2002). If the fitness loss is so severe that it leads to further population declines, the population could get caught in an extinction vortex (Fagan & Holmes, 2006; Gilpin & Soulé, 1986). To counter these challenges, genetic rescue has been proposed as a conservation management strategy, in which human‐assisted migration is used to stimulate gene flow between disconnected populations. This, in turn, is anticipated to introduce new alleles, which may help reduce the realized load, and potentially increase the evolutionary potential (Frankham et al., 2002; Jackson et al., 2022; Tallmon et al., 2004; Whiteley et al., 2015).

Although inbreeding depression is challenging to both identify and quantify in wild populations, its identification is critical to the management of threatened populations (Grueber et al., 2010; Pemberton, 2008). Traditionally, inbreeding has been studied by building pedigrees, which requires complete generational information that is often incomplete even in well‐monitored natural populations. A further challenge relates to the distinct effects of inbreeding depression that are associated with different life stages (Grueber et al., 2010; Szulkin et al., 2007) – for example, embryonic lethal recessive mutations cannot be identified if embryos are difficult to locate or access, while quantifying lifetime reproductive success is difficult in species that exhibit extra‐pair maternity/paternity. Therefore, as good quality, long‐term monitoring data for traits are often lacking for wild populations, inbreeding is increasingly quantified with genomic data alone (Kardos et al., 2016; Pierson et al., 2016).

The Seychelles magpie‐robin (*Copsychus sechellarum*, hereafter referred to as SMR) is a small passerine bird endemic to Seychelles. Although the SMR is thought to have once inhabited all ~40 of the granitic islands of Seychelles, the settlement of the archipelago by humans in 1770 triggered the collapse of the SMR populations. The species became listed as Critically Endangered on the IUCN Red List in 1994. As has often occurred following human invasion, the native habitat was not only destroyed in favour of plantations, but alien predators such as ship rats (*Rattus rattus)* were introduced, leading to much of the native wildlife being displaced or destroyed (Cheke, 2010; Stoddart, 1970). As by 1965, the entire SMR population had contracted to fewer than a dozen individuals on a single island (Fregate Island). A comprehensive recovery initiative was initiated in the 1990s, which included a series of translocations between 1994 and 2008 to find new populations on four other islands. In addition, the population recovery was helped by native habitat restoration and management, stringent adherence to biosecurity protocols, comprehensive studies of invertebrate populations, provision of supplementary feeding to ensure adequate nourishment, installation of water stations to mitigate water scarcity during dry spells (Bristol et al., 2005; Burt et al., 2016) and provision of medical care in instances of injury or illness (S.M.A.R.T. pers. comm.).

**FIGURE 1:**
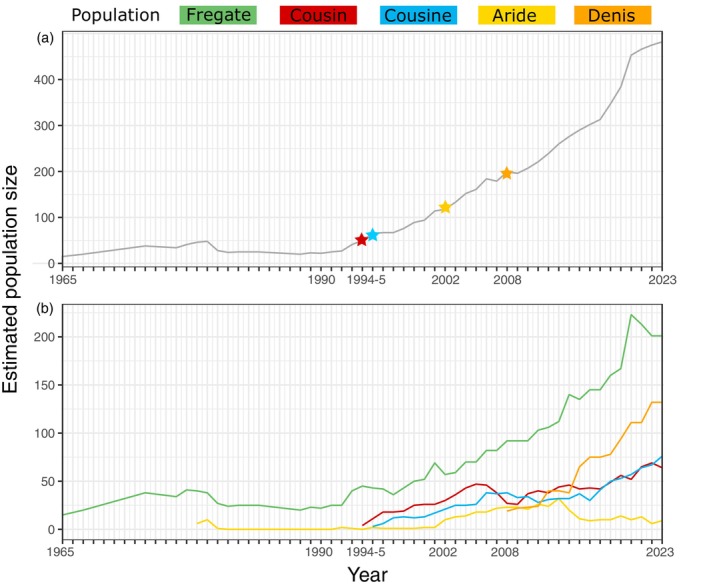
SMR population trends 1965–2023. (a) Cumulative census population size estimates across all islands. Receiving island coloured by field band colour: Cousin (red) received a total of six birds; Cousine (blue) 9; Aride (yellow) 15; Denis (orange) 20, and stars indicate the date of first translocation (b) Cumulative population sizes of each island. All estimates are the ‘maximum recorded’ estimate per year. Population size data was compiled from Dawson, 1965; Gaymer et al., 1969; Burt et al., 2016 and unpublished data provided by the Seychelles MAgpie‐robin Recovery Team (S.M.A.R.T.). Where some data are ‘missing’ or not up to date, the estimates of the previous known records are used.

Thanks to these measures, today the census population stands at ca. 500 individuals inhabiting five islands (Figure 1a) with most islands seeing a continual increase (Figure 1b), and the SMR has been downlisted to Endangered since 2005. While this offers some hope for the species' survival, valid concerns exist as to whether the original population bottleneck and subsequent translocations have consequences for the long‐term viability of the species.

At the start of the SMR recovery program, nearly half of all eggs were thought to be infertile given they were not viable at 10–14 days, an observation Hockey (1997) proposed to be phenotypic evidence of inbreeding depression. Despite this, the SMR has experienced continuous population growth over the past ~30 years (Figure 1a). We previously identified that the current SMR population is characterized by very low levels of genetic diversity and long identical‐by‐descent tracts (Cavill et al., 2022). Despite such genomic erosion, the population size increased, most likely helped by effective conservation management. Indeed, the severity of inbreeding depression depends on the level of inbreeding and the amount of masked load (Bertorelle et al., 2022). Therefore, genetic assessments are important for effective conservation action and assure the SMR's long‐term survival.

Museum voucher specimens can be invaluable for answering such questions, as the genetic data we obtain from them offer a historical baseline with which to quantify that which has been lost (Díez‐Del‐Molino et al., 2018). These temporal genomic analyses allow us to assess whether genetic diversity has decreased, and whether the genetic load has been purged or accumulated during these demographic changes. When temporal genomic analyses are further combined with individual‐based simulations, a species' extinction risk and long‐term viability can also be assessed (e.g. Dussex et al., 2023; Kyriazis et al., 2023).

In this study, we assessed the population history of the SMR using genomic tools coupled with field‐based monitoring data, in order to reconstruct the story of their past, present and future. Specifically, we combined new genomic data generated from historical SMR samples with previously described data from modern SMR populations (Cavill et al., 2022). We, thus, characterized the extent of genomic erosion. Our total dataset consists of 201 re‐sequenced SMR genomes that span ~150 years, with the 37 historical genomes derived from museum specimens collected across five islands and dating to between 1861 and 1991. We used genetic parameters to assess temporal and demographic change in genetic diversity across this temporal sampling window, which follows the species' decline that started shortly after human colonization, through the known historical bottleneck, and extends into the recent conservation translocations. Given we know that natural migration between the populations on the different islands is currently extremely limited (Burt et al., 2016), we further estimated whether genetic rescue could curb the putative effects of ongoing inbreeding in the SMR, in order to provide a robust recovery plan for the future. We lastly present simulated scenarios of genetic rescue attempts between the extant populations, to explore how different forms of action could alter the future trajectory of the SMR's genomic erosion. This sets the stage for forward‐looking strategies that could involve a proactive recovery plan to address the SMR's future genetic and conservation challenges.

## 2 MATERIALS AND METHODS

### 2.1 Sample collection, DNA extraction, library construction and sequencing

Our historical SMR dataset was derived from 37 toepad samples provided by the American Museum of Natural History, New York (*n* = 9); University Museum of Zoology, Cambridge University (*n* = 5); Museum of Comparative Zoology, Harvard University (*n* = 1); Natural History Museum, London (*n* = 14); Muséum d'Histoire Naturelle, Paris (*n* = 4); National Museum of Natural History, Smithsonian Institution (*n* = 2); and The Peabody Museum of Natural History, Yale University (*n* = 2). These samples were originally collected at timepoints described as ‘pre‐1861’ to 1991, from four of the original island populations, and one translocated population (Suppl. Table S1). Historical samples were processed in a PCR‐free laboratory dedicated to handling ancient DNA. DNA was extracted following the Campos and Gilbert (2012) extraction for keratinous materials, although incorporating a modified purification based on Monarch DNA clean‐up columns (New England Biolabs). Genomic libraries were built and purified following the SCR single‐stranded DNA protocol (Kapp et al., 2021), and modified to use adaptors compatible with BGIseq technology (Van Grouw et al., 2023). Libraries were PCR amplified and uniquely indexed in triplicate using a PFU Turbo CX recipe (as in Cavill et al., 2021) which allows for the incorporation of uracil bases where DNA damage has occurred. The amplified libraries were pooled and sequenced using the BGIseq4000 platform, using either 100 bp single‐end or 100 bp paired‐end read chemistry, yielding between ca. 10 M and 418 M reads per sample.

The modern dataset consists of 141 previously published SMR genomes (BioProject number PRJNA722144, Cavill et al., 2022) plus 23 newly re‐sequenced SMR genomes. DNA for these additional modern samples was extracted following the DNeasy protocol for blood and tissue (Qiagen) and fragmented using COVARIS LE220‐plus to shear the DNA to ~350 bp. SCR libraries were built on the fragmented DNA as outlined above, and were PCR amplified with TaqGold (see Cavill et al., 2022) to incorporate BGIseq indices, then sequenced as a pool on a single 150PE BGIseq4000 lane, yielding ca. 14 M and 108 M reads per sample.

### 2.2 Mapping of raw reads

Raw sequencing reads were mapped to the published SMR nuclear and mitochondrial reference draft genome assemblies (Feng et al., 2020, BioProject number PRJNA545868) using the PALEOMIX 1.3.6 BAM pipeline (Schubert et al., 2014). At this step, sequences from modern samples were mapped using BWA‐MEM (Li, 2013) following default filtering parameters, while sequences from historical samples were mapped using BWA‐BACKTRACK (Li & Durbin, 2009) following default parameters and a shorter read length allowed (25 bp). PCR duplicates were removed with the Picard Toolkit (Broad Institute, 2019). We further removed sex‐linked scaffolds using a coverage‐based method in ANGSD v.0.935 (Korneliussen et al., 2014), and repetitive regions (Feng et al., 2020) using samtools/v1.9 ‐view (Danecek et al., 2021). The resulting bam files were used as input for most analyses. Transitions were excluded from subsequent analyses to ensure that cytosine modifications derived from DNA damage did not bias results.

### 2.3 Estimating historical effective population size

We used GONE (Santiago et al., 2020) to estimate historical effective population size (Ne) over 600 years (166 generations) into the past. To compensate for the very low genetic diversity exhibited in the modern SMR, restricting the LD signal necessary for the reconstruction of past demography, we carried out this analysis on subsets of both the historical and modern datasets. We selected nine samples from our Marianne Island dataset, all of which were sequenced to a minimum of 4X coverage, and were collected within a short timeframe (~1880–1900). This population is of particular interest as in contrast to the other inner islands at this time that had already been heavily affected by invasive species like rats and cats, its birdlife was still notably abundant (Cheke, 2010) (although not untouched, as Newton (1867) observed that in 1865 its SMR population was already in decline). To represent the modern SMR, we used the Fregate Island population, which founded the translocated populations. For both datasets, we generated separate PLINK tped files for scaffolds above 10 Mb (total 728 Mb) with ANGSD (using commands ‐doMajorMinor 1 ‐doGeno −4 ‐doPlink 2 ‐doMaf 1 ‐remove_bads 1 ‐uniqueOnly 1 ‐rmTrans 1 ‐SNP_pval 1e‐6 ‐minMapQ 30 ‐minQ 20 ‐doCounts 1 ‐doDepth 1 ‐BAQ 2, ‐C50, and the SMR draft genome assembly as the reference genome). We then used PLINK to recode the resulting tped and tmap files to ped and map files, which were used as input for GONE. As the genetic distance between markers is unknown, we adopted the recombination rate of 2 cM/Mb (centimorgans per megabase) based on the zebra finch (*Taeniopygia guttata*) estimates presented in Backström et al. (2010). The results presented are the respective means of 40 replicates per temporal population. To put the results into the context of our settlement timeline (~250 years ago), we use the estimated generation time for the species of 3.6 years (Birdlife International, 2017). All results presented throughout the text are visualized with the ggplot2 v3.3.3 package (Wickham, 2016) in R (R Core Team, 2022) unless otherwise stated.

### 2.4 Genetic population assignment and ancestral components of the historical SMR

We used a combination of three methods to explore the historical population structure and confirm, assign or reassign the documented collection origin of the historical samples: principal component analysis (PCA), admixture and phylogenetics. We first used ANGSD v.0.935 to generate two beagle files (‐doGlf 2) containing autosomal genotype likelihoods for SNPs: one for the historical dataset and one for the combined modern and historical dataset. We used filtering parameters: ‐doMajorMinor 1 ‐doPost 1 ‐doGeno 5 ‐doMaf 1 ‐remove_bads 1 ‐uniqueOnly 1 ‐rmTrans 1 ‐SNP_pval 1e‐6 ‐minMapQ 30 ‐minQ 20 ‐doCounts 1 ‐doDepth 1 ‐BAQ 2, ‐C50, which identified 1,413,970 and 1,420,406 transversion variable sites in the historical and combined datasets respectively. These genotype likelihood files were used as input for PCAngsd v0.95 (Meisner & Albrechtsen, 2018) to generate two pairwise covariance matrices (one historical and one combined). We then used the historical genotype likelihood file as input for NGSadmix version 1.0.0 (Skotte et al., 2013) to assess ancestral proportions of our historical dataset, for comparison to the results previously established for the modern SMR (Cavill et al., 2022). In NGSadmix, we ran up to five ancestral components (k) and repeated the analysis up to 100 times. If at least two of the best runs produced the same likelihoods, they were considered to have converged. We present results from *k* = 2 and *k* = 4 in the main text, and the full plot in Figure S1.

We generated a distance matrix using ANGSD, using the genome of a Karoo scrub robin *Cercotrichas coryphaeus* (Feng et al., 2020, BioSample no. SAMN12253994) as an outgroup, and applying the same filtering parameters as described above, but with additional options ‐doIBS 2 ‐makeMatrix 1 ‐minminor 2. The matrix was read into FastME 2.0 to create a distance‐based phylogeny (Lefort et al., 2015). We used FigTree v1.4.4 (Rambaut, 2018) to visualize the results.

### 2.5 Assessing genetic variation of modern and historical samples

We estimated autosomal heterozygosity to quantify genetic variation and to compare changes in genetic variation across sampling time points. We first generated whole‐genome site allele frequency (‐doSaf) files in ANGSD v0.935, using samples with a depth of coverage above 4X and the filtering parameters described above. The files were then used as input for realSFS (ANGSD v0.921) to generate 100 bootstrapped estimates for genome‐wide heterozygosity, and the average value was calculated from the bootstraps for each sample. We further explored regions of lost and conserved heterozygosity through a temporal comparison of three Fregate Island samples with similar coverage (1861, 1958, 2015), by estimating the individual SFS in 50 Kb windows with 10 Kb steps, using scaffolds larger than 10 Mb.

### 2.6 Estimates of inbreeding in historical populations

We identified and characterized runs of homozygosity (ROH) in the SMR in a temporal comparative analysis. Treating the historical and modern datasets separately, we generated ped files for scaffolds larger than 10 Mb using the same filtering parameters for GONE. We identified ROH with PLINK 1.9 (Chang et al., 2015) using the following parameters in order to obtain a reliable comparison of ROH (specifically F_ROH_) between the historical and modern populations: ‐‐homozyg‐snp 50 ‐‐homozyg‐kb 300 ‐‐homozyg‐density 50 ‐‐homozyg‐gap 1000 ‐‐homozyg‐window‐snp 50 ‐‐homozyg‐window‐het 5 ‐‐homozyg‐window‐missing 20 ‐‐homozyg‐window‐threshold 0.05. F_ROH_ was defined as the proportion of the utilized genome (728 Mb) in ROH, with ROH defined above 1 Mb in length. To estimate generations since inbreeding events, we followed g = 100/(2rL), where r is the recombination rate (2 cM/Mb), and L is the length of the ROH in Mb (Thompson, 2013). We estimated the time since an inbreeding event for different ROH lengths, establishing that a length of 1 Mb indicates inbreeding ~33 generations ago, 5 Mb as ~6 generations ago, while 16 and 30 Mb are ~2 and ~ 1 generations respectively.

### 2.7 Genetic load analyses with SnpEff and phyloP

#### 2.7.1 The ancestral state of the SMR genome

**FIGURE 2:**
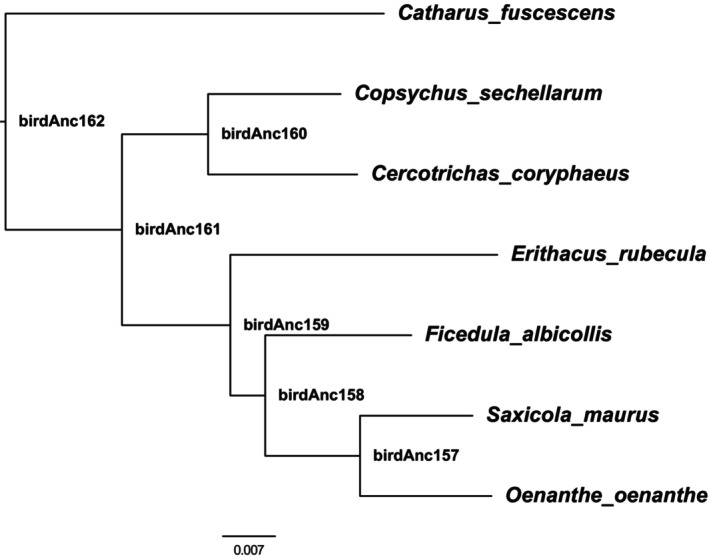
A reconstructed ancestral node. A phylogenetic tree inferring the ancestral state based on a phylogenetic tree of multiple closely related species.

To polarize our SNPs as ancestral or derived, the ancestral state of the SMR genome was generated from the B10K genome alignment of 363 species (Feng et al., 2020). The ancestral state of each node is available at https://cgl.gi.ucsc.edu/data/cactus/363‐avian‐2020.hal. We consider the reconstructed ancestral node (birdAnc161, Figure 2) of a clade composed of the SMR and five other species (*Copsychus sechellarum*, *Cercotrichas coryphaeus*), (*Erithacus rubecula*, (*Oenanthe*, *Saxicola maurus*), *Ficedula_albicollis*) that diverged ~19 MYA (Kumar et al., 2022) as the ancestral state, and extracted the fasta from the alignment using *hal2fasta* as implemented in hal v2.2 (Hickey et al., 2013). We subsequently only included the mutations classified as derived relative to this ancestral state in our analyses. Such derived variants fall into one of three categories: (1) harmful variants that contribute to the genetic load, (2) selectively neutral variants that diverge between species due to genetic drift or (3) beneficial variants that constitute species‐specific adaptations.

#### 2.7.2 Estimating the genetic load

To assess the putative impact that the bottleneck, inbreeding and genetic drift have had on the fitness of the modern SMR, we predicted the deleterious effects of mutations with SnpEff (Cingolani et al., 2012) and phyloP scores (Hubisz et al., 2011). First, we used GATK HaplotypeCaller v3.6 to create single‐sample gVCF from bam files, and standard filtering parameters (‐‐emitRefConfidence BP_resolution ‐variant_index_type LINEAR ‐variant_index_parameter 128,000 ‐stand_emit_conf 30 ‐stand_call_conf 30 ‐‐min_base_quality_score 20 ‐mmq 30). Resulting gVCFs were combined, genotyped and then filtered with GATK ‘best practice hard filtering’ (Van der Auwera et al., 2013). We annotated the VCFs using SnpEff v4.3T (Cingolani et al., 2012), and by adding the SMR reference genome fasta file and annotation gff file (Feng et al., 2020) to the SnpEff database. SnpEff classifies SNPs as either loss‐of‐function (LoF), missense or synonymous, identifies whether the mutations lie in coding or non‐coding sequences, and provides a predicted level of impact on the resulting protein (high, moderate or low). To prevent the introduction of biases imposed by sequencing errors derived from post‐mortem DNA damage, and/or the use of low coverage in the historical data, the final variant list was filtered to remove singletons and transitions, and any samples with an overall depth of coverage of less than 5X. Genotyped sites were filtered to retain those at higher than 5X overall depth, and heterozygous sites with higher than 3X per allele. While filtering out singletons reduces the risk of including sequencing artefacts, we recognize it also removes rare mutations from the analysis.

We assessed changes in genetic load over time to monitor the impact of population size decline (and recovery) on individual fitness and population viability. In particular, we assessed the amount of genetic load that has no immediate effect on fitness, that is, the masked load (or inbreeding load), and the part of genetic load that reduces fitness, that is, the realized load (Bertorelle et al., 2022). Here, we assume that heterozygous deleterious mutations that are (partially) recessive form part of the masked load (Bertorelle et al., 2022; Dussex et al., 2023; Smeds & Ellegren, 2023). If they are completely recessive (dominance coefficient, *h* = 0), such heterozygous mutations do not reduce fitness. Homozygous deleterious mutations are assumed to constitute the realized load. However, mutations that are partially dominant (*h* > 0) do reduce fitness also as heterozygotes and contribute to the realized load. Nevertheless, in this study, we simply defined the masked load as the heterozygous genetic load, and the realized load as the homozygous genetic load.

During population size declines, some mutations become homozygous due to inbreeding and genetic drift, which increases the realized load (Bertorelle et al., 2022; Pinto et al., 2024). This increase in realized load is equivalent to the reduction in the masked load, and results in inbreeding depression (Bertorelle et al., 2022; Dussex et al., 2023; Sachdeva et al., 2022). However, because we do not know the dominance coefficients (*h*) of mutations, we estimated the masked load by counting the number of high‐ and moderate‐impact heterozygous derived alleles for missense and LOF mutations. Furthermore, we estimated the realized load by counting the homozygous alleles. We normalized LOF and missense derived allele counts over synonymous counts to ensure comparability across the historical and modern data (as in van der Valk et al., 2019; Dussex et al., 2021). The effects of this normalization (i.e. direct counts vs. relative counts of genetic load) are detailed in Figure S2.

We recognize that both the filtering parameters, and the method of calculating relative counts described above, reduce the statistical power to detect purging and loss of deleterious variants (through purifying selection and drift respectively). This is an inherent limitation of historical datasets; to compensate for DNA degradation and the variability in data coverage and missingness between samples, historical data must be filtered, and the genetic load results should be presented as a ratio between historical and modern estimates. Our estimates of purging and drift are, therefore, likely conservative as we would have missed many rare variants in the historical samples. Nevertheless, it is important to note that the vast majority of rare variation (deleterious or not) would likely be lost through a bottleneck as severe as that experienced by the SMR. Hence, we argue that our focus on the dynamics of more common deleterious variants that survived the bottleneck is justified, and that it is a robust approach that comes with a downside.

We next used conservation scores to assess the variation in the genome for evolutionary stability and biological function. We utilized phyloP scores generated from the B10K 363‐way avian species alignment (including the SMR), and restricted our analysis to loci identified as significantly conserved (Feng et al., 2020). We subsequently used hal v2.2 (Hickey et al., 2013) to lift over the score to our SMR reference genome. A total of 273,307 SNPs derived from the ancestral state were included in the analysis after liftover and conservation filters. Genetic load estimates for each individual were calculated as the weighted sum of the phyloP scores multiplied by the number of derived alleles, and normalized by the total number of SNPs for each individual. To facilitate the interpretation of the modified phyloP scores, we consider the results against the lowest significant score for the B10K 363‐way dataset of ≥1.957. We acknowledge that phyloP scores (and other mutation‐impact scores) do not directly translate into fitness effects or selection coefficients, and that the function between these scores and fitness is unlikely to be linear. Nevertheless, the approach of summing mutation‐impact scores used in this study is commonly performed in other studies, and it provides a preliminary estimate of the genetic load.

### 2.8 Simulating genetic rescue

We performed individual‐based forward simulations with SLiM 3.6 (Haller & Messer, 2019) to examine the impact of different interventions for the purpose of genetic rescue in the SMR populations. First, we recreated the genetic diversity loss over the SMR population collapse and recovery (Figure 8a) and confirmed that our simulations reflect the ~3‐fold loss of genetic diversity observed in the empirical data (Figure 8b) and the increase in realized genetic load (Figure 8c). Next, we tested alternative scenarios for genetic rescue in the recovered, translocated populations. We consider two alternative receiving populations, a large population (Fregate Island) and a smaller population (Cousin Island). Each of these alternative populations received migrants from each of the other populations, at different amounts and frequencies to represent different genetic rescue scenarios; (1) single/low: a single translocation event of one effective migrant per source population; (2) single/high: a single event of five migrants per source population; (3) sustained/low: one event every 10 years of one migrant per source population; (4) sustained/high: one event every 10 years of five migrants per source population. The sustained scenarios had seven translocation events over 70 years (up to the year 2100). We further tested the impact of selecting donor individuals either at random, or those with the highest nucleotide diversity or those with the lowest realized genetic load. The scenarios of intervention for genetic rescue were compared to a scenario with no intervention (i.e. do‐nothing approach; Ralls et al., 2018).

The simulated demographic trajectory was informed by the ancestral population size inferred with GONE (see above), the known and estimated island‐carrying capacities, and the recorded census trajectories from 1965 to 2022. The trajectories were maintained for another 100 years (to 2120) to simulate future dynamics. The simulations incorporated ecological parameters such as reproductive age of one, fecundity (Figure S3a), lifetime monogamy and mortality probabilities (Figure S3b). The combination of these parameters resulted in an average generation time of 3.6 simulation steps, consistent with the generation time of the SMR. Therefore, we can assume that each simulation step is approximately 1 year, thus presenting the results in a per‐year scale. We simulated an exome composed of 20,000 genes of 500 bp each with a recombination rate *r* = 1e^−4^ (no recombination within genes), and a per base mutation rate *m* = 3e^−8^. Deleterious mutations were sampled from a gamma distribution (mean = −0.05; shape = 0.5), and a negative dominance (*h*) and selection coefficient (*s*) relationship. We have shown before that these distributions of *s*, and the *sh* relationship, resemble naturally emergent frequency distributions. That is, non‐recessive mutations that are mildly to highly deleterious are effectively removed by purifying selection and are rare in populations. In addition, we have also shown that alternative s and h distributions do not substantially affect the interpretations regarding the dynamics of the genetic load in response to population collapse (for more details, see Dussex et al., 2023).

## 3 RESULTS

We generated 37 historical genomes to a depth of coverage of 0.17–10.73X, 29 of which were above 4X. These historical genomes represent five historical island populations, dating from pre‐1861 (100 years before the documented bottleneck) to 1991 (the beginning of the SMR recovery program). We used these historical genomes to set a baseline for the genetic history of the SMR, and to perform comparative temporal genomic analyses using the 164 contemporary genomes.

### 3.1 Historical population structure resolves the origin island of the oldest SMR sample

**FIGURE 3:**
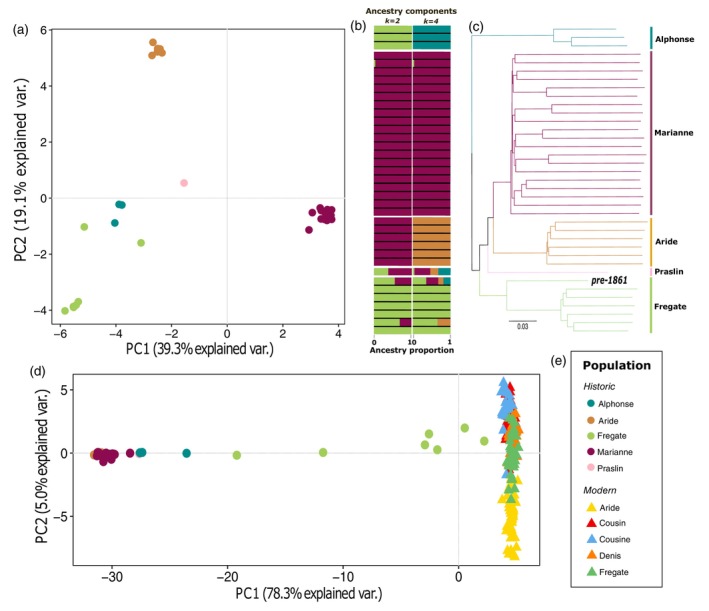
Multi‐panel structure exploration of the historical and modern SMR. (a) PCA based on 1,413,970 transversion variable sites identified in the historical dataset. PCs 1 and 2 explain 58.4% of variability between samples. (b) Admixture plot assuming 2 and 4 ancestry components where each bar represents the ancestry proportions per sample. The order of samples aligns with that in (c). (c) Whole‐genome distance‐based phylogeny inference using all 37 historical samples. Island groups are highlighted by colour‐coded vertical bars (right) and are annotated with the island name, the position of the oldest historical sample is highlighted in bold typeface. (d) PCA created using combined historical and modern datasets and a total of 1,420,406 transversion variable sites. PC1 explains the majority (78.3%) of the variance observed. (e) Legend.

The PCA for the historical dataset (Figure 3a) shows the majority (58.4%) of the variance between samples can be explained through the first two principal components (PCs). Clear clusters are distinguished for Aride, Marianne and the Fregate Island samples collected around or after the documented bottleneck, which allowed us to identify potentially mislabelled samples, and assign locality to those which were previously unknown (Suppl. Table S1). The admixture analysis assuming two ancestral components (*k* = 2) revealed a component predominantly shared between the Fregate Island population and the translocated Alphonse population (Figure 3b). This provides insight into the previously ambiguous cluster involving Alphonse and pre‐bottleneck Fregate Island samples. Assuming four ancestral components (*k* = 4), we observed a distinct population structure, with notable admixture in both the oldest samples that date to before 1861, and those from the larger mainland population of Praslin. The phylogenetic analysis revealed a distinct island‐related population structure. This enabled us to assign the pre‐1861 sample to the Fregate Island population (Figure 3c), and use it as such in all subsequent analyses. The observed variance within the historical Fregate Island population in the structure analyses seems to stem from the timing of sample collection. The PCA, conducted on both historical and modern datasets, demonstrates significant temporal genetic differentiation, with nearly 80% of the variance accounted for by the first principal component (PC1; Figure 3d). The cluster of historical Fregate Island samples that are placed directly before the modern group represent some of the few remaining SMR in the 1950s, approximately reflecting the timeline of sample collection. The modern populations, that were founded from this last population, are structured along the PC2 axis, reflecting a history of genetic drift and inbreeding following the translocations.

### 3.2 A history of declining population size and low genetic diversity

**FIGURE 4:**
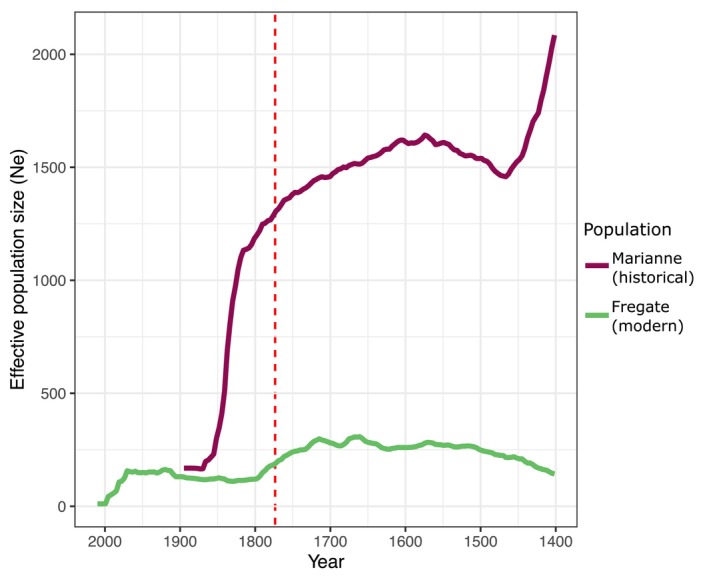
Historical effective population size reconstruction using samples from the historical Marianne Island population (*n* = 10) and from the modern Fregate Island population (*n* = 31) identifies the known bottleneck of the SMR. The date of human settlement is ~250 years (~69 generations) ago and is indicated by the horizontal red dashed line.

Our historical effective population size estimates indicate that human settlement of the Seychelles, an estimated 250 years (69 generations) before sample collection, coincides with the start of the species' known bottleneck (Figure 4).

**FIGURE 5:**
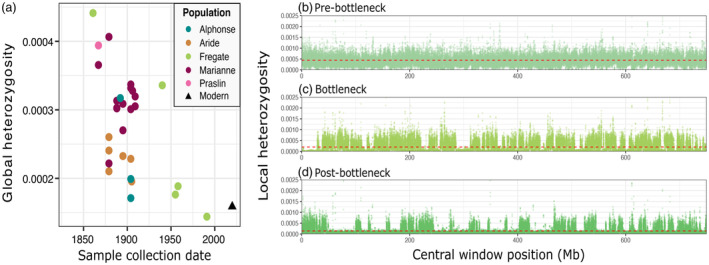
A 3‐fold loss of heterozygosity over 150 years. (a) Autosomal global heterozygosity estimates generated with ANGSD for each individual historical sample, and the average heterozygosity estimate for the ‘Modern’ dataset taken from Cavill et al. (2022) for comparison. (b) Local heterozygosity across the genome for a Fregate Island sample collected prior to 1961 (genome‐wide coverage 10X). The dashed red line indicates individual average global heterozygosity estimate. (c) As (b) with a sample collected in 1958, around the time of the documented bottleneck (8X coverage). (d) as (b) with a modern Fregate Island sample collected in 2015 (8X coverage).

Genome‐wide autosomal heterozygosity estimates range from 0.00044 in our oldest Fregate Island sample, and decrease along a gradient to 0.00015, calculated as the average from all modern samples (Figure 5a). This reveals a significant association between sample collection date and heterozygosity (*r*_s_ = −0.45514, *p* = 0.0115). Additionally, the stark contrast in heterozygosity between islands that were sampled around the same time underscores the impact of stochastic processes on the remaining individuals. We draw attention to the Fregate Island population (Figure 5b–d), which did not suffer extirpation and serve as the founder population for all modern populations. While the historical heterozygosity estimates are low compared to those obtained from museum samples of other *Copsychus* species (Ng et al., 2017; Wu et al., 2022), heterozygosity is evenly distributed across the genome in our oldest pre‐bottleneck sample (Figure 5b). In contrast, the sample from the bottleneck period reveals extended regions of reduced heterozygosity (Figure 5c), and these regions are further extended along the genome in the post‐bottleneck sample (Figure 5d).

### 3.3 An accumulation of deleterious mutations in the modern SMR

**FIGURE 6:**
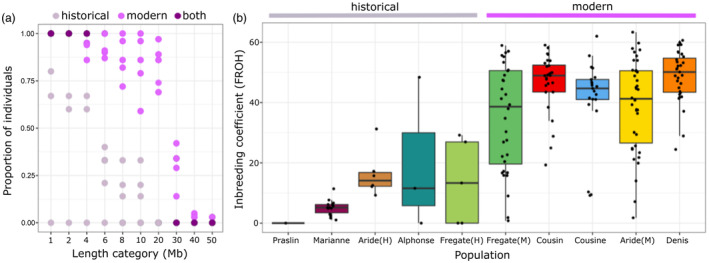
Increased inbreeding in the modern SMR populations. (a) Proportion of historical and modern populations of ROH length categories between 1 Mb and 50 Mb. Individual points represent island populations, and overlapping historical and modern data points are coloured in dark. (b) F_ROH_ by island population calculated as the proportion of genome in ROH above 1 Mb. Individual points represent single individuals, and the horizontal line inside each box represents the population average. Modern populations are presented in order of the date of translocation.

We observe an overall increase in the length of ROH in the modern populations (Figure 6a), which culminates in an increased F_ROH_ (genomic inbreeding coefficient) in the modern samples (Figure 6b). Historical samples that harbour at least one very long ROH, leading to an overall higher F_ROH_, are predominantly from introduced (Alphonse) or bottlenecked (Fregate Island) populations. All modern populations have at least one individual with an ROH of up to 30 Mb, which we estimate to be the result of inbreeding in the last generation. These estimates are consistent with recent monitoring data that reveals the prevalence of offspring from consanguineous matings (S.M.A.R.T., *unpublished data*).

**FIGURE 7:**
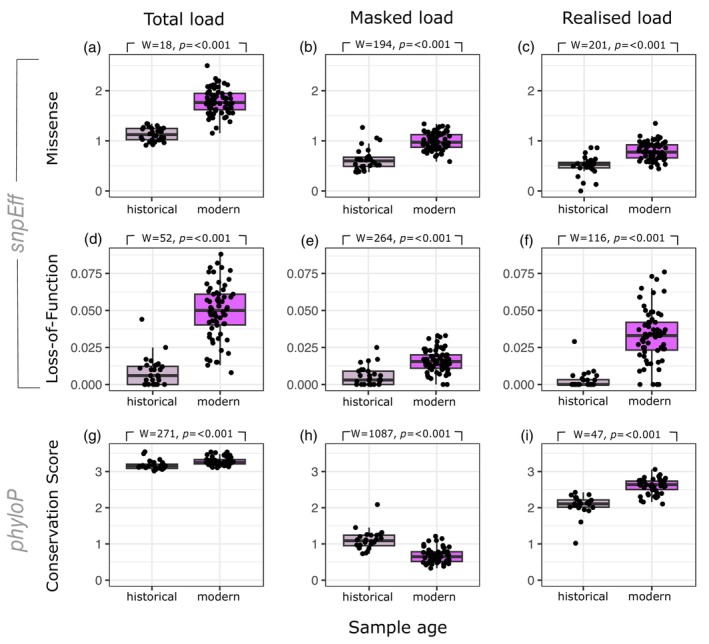
Burden of genetic load is significantly* higher in modern populations. (a) Relative total genetic load is the combined allele counts of high and moderate impact, heterozygous and homozygous mutations (normalized over total synonymous mutations) resulting in a non‐synonymous (missense) change in the resulting amino acid sequence. (b) and (c) as (a) but heterozygous derived mutations (masked load) and homozygous derived mutations (realized load) respectively. (d–f) as (a–c) but mutations result in a nonsense (Loss‐of‐Function) change in the amino acid sequence. (g) Conservation scores calculated as weighted sum of phyloP score * number of heterozygous + homozygous derived alleles/total number of SNPs. (h) and (i) as (g) but heterozygous derived alleles and homozygous alleles only respectively. *Results of Wilcoxon rank sum tests presented above each respective panel.

The total genetic load is higher in the modern samples compared to the historical samples, and this pattern is found in missense mutations, LOF mutations and conservation scores (Figure 7a,d,g). Surprisingly, both the relative masked load and realized load of missense mutations and LOF mutations appear to have increased in modern samples (Figure 7b,c,e,f) which we have unpacked with the support of simulations in Suppl. Figure S3. However, when expressed in conservation scores, the masked load is higher in the historical samples than in the modern samples (Figure 7h), which is consistent with predictions based on evolutionary genetic theory (Bertorelle et al., 2022). We further observe that the realized load is highest in the modern samples (Figure 7i) which is also consistent with theoretical expectations. Although we do not directly link conservation scores to selection, we recognize that high scores are likely to reflect deleterious variants in the SMR. Given that these variants are highly conserved across hundreds of bird species, it is likely that derived variants have a negative effect on fitness.

### 3.4 Genomic erosion dynamics with and without translocation intervention

**FIGURE 8:**
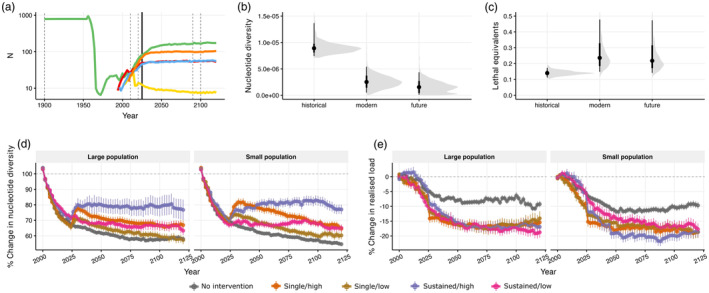
Genetic rescue is a promising tool for SMR conservation. (a) SMR demographic decline and recovery: 1965–2020 population size utilized empirical data, future trajectory is simulated. The horizontal dashed lines are the ‘modern’ and ‘future’ time slices for panels (b) and (c) under the ‘no intervention’ scenario, and the solid line represents the time of genetic rescue for (d) and (e). (b) and (c) show nucleotide diversity and realized load across all populations and replicates under the ‘no intervention’ scenario. (d) and (e) show nucleotide diversity and realized load trends relative to the year 2000 for the ‘no intervention’ and all the alternative attempted genetic rescue scenarios, picking individuals at random.

Our simulations revealed that while a single translocation event of only a few individuals results in a brief increase in nucleotide diversity in all intervention scenarios, ultimately diversity continues to decline over the long term (Figure 8d). The genetic diversity increase observed in all scenarios is likely due to the immediate mixing of genetic diversity between populations. In contrast, for the sustained/high scenario, we observed a continued increase in all intervention scenarios, and we observed a similar pattern when considering genetic load (Figure 8e). For the sustained/high scenarios, the realized load is roughly halved when compared to no intervention. While in general, we found that any intervention strategy was better than no intervention, our results demonstrate that the benefit of the intervention is greater when multiple translocation events are performed over several decades, and when using a larger number of individuals each time. In these simulated scenarios, there is little discernible difference in the SMR between random donor selection and targeted donor selection (Figure S4). Notably, while some interventions have clear positive effects on genetic diversity and genetic load, none of the scenarios tested were able to restore the ancestral levels before the bottleneck (Figure S5).

## 4 DISCUSSION

The Seychelles magpie‐robin (SMR) underwent a severe and well‐documented population bottleneck leading up to the 1960s, at which point the species was perched on the edge of extinction. Our analyses confirm that the effective population size (Ne) declined sharply and severely, shortly after human settlement. The Seychelles islands were not isolated from humans before European settlement; there is both anecdotal and other evidence of people (including pirates) visiting the Indian Ocean and Seychelles before the late‐1700s colonization (Barbour, 2008; Jourdain & Foster, 1967). Pre‐settlement voyages have been known to introduce invasive alien species in other parts of the world, resulting in population decline or extinction of endemic island species (Wood et al., 2017). Such early introductions could potentially account for the observed decline over the centuries preceding the colonization of Seychelles. More recently, translocations have allowed the species to recover from a single population of circa 20–30 individuals at the start of the recovery program, to around 500 individuals distributed across five islands. We hypothesized that these translocations may have created further genetic bottlenecks, and we explored the effects of this human‐assisted demographic recovery on the species' genetics. To examine the overall genomic consequences of the severe bottleneck on the contemporary SMR populations, we generated the necessary baseline data by resequencing the genomes of museum specimens, allowing us to assess temporal genetic changes. We combined our genomic results with the documented evidence of the SMR history to enhance our understanding of the species' historical narrative and its potential for long‐term viability. Ultimately, our aim was to provide insights that might help guide future conservation efforts, with a focus on intraspecific genetic rescue.

### 4.1 Relaxed selection from intense environment management

The SMR recovery program has certainly been a great success in terms of demographic recovery, with the resulting increase in its geographical range and population size reducing the extinction risk it faced just 30 years ago. Through this recovery process, a heavily controlled environment has been created for the SMR. Although the current environment is undoubtedly different from the natural environment in which the species would have existed in the past, this change has been necessary given the contemporary challenges they face. Our results, however, show that the SMR population increased its genetic load during its demographic recovery. The increase in its realized load is particularly concerning because it likely results in the expression of the harmful fitness effects of recessive deleterious mutations. Although inbreeding depression may have reduced the SMR's fitness and population viability, it appears that the intense local management efforts may have relaxed purifying selection. This, in turn, would have assisted population recovery, but may also have elevated the genetic load by reducing the efficacy of selection. Furthermore, their maintenance in an environment with high biosecurity measures, which keeps the islands largely free from predators or resource competitors, and ensures a low risk of disease being introduced, may have also enabled a resulting build‐up of deleterious mutations in their genomes. This genetic load might have been purged from the population under more challenging (natural) conditions. Conservation management (e.g. supplementary feeding) reduces resource competition, which helps accelerate demographic recovery. However, in such expanding populations, soft selection becomes less efficient, as the competition between individuals relaxes. In addition, supplementary feeding may also reduce the efficacy of hard selection by increasing juvenile survival. This unintended consequence of conservation management is probably not unique to the SMR, but may be common in other heavily‐managed species, such as the pink pigeon *Nesoenas mayeri* (Jackson et al., 2022), the crested ibis *Nipponia nippon* (Feng et al., 2019) and the island fox *Urocyon littoralis* (Robinson et al., 2016), and has also been proposed for domestic crops and animals (Makino et al., 2018; Renaut & Rieseberg, 2015).

### 4.2 The promise and pitfalls of genetic rescue

Although genetic rescue can be an important tool in the conservation of endangered species, it is rarely used due to the risks associated with ‘outbreeding’ depression (Fitzpatrick et al., 2023; Waller, 2015). However, the risks should be always compared against a scenario of doing nothing (Ralls et al., 2018). Our simulations support the idea that without intervention, the SMR will likely continue along the same trajectory of genetic diversity loss and accumulation of overall genetic load. The species may, therefore, benefit from future translocations to facilitate genetic rescue. In an ideal scenario, genetic rescue is achieved through gene flow from a genetically diverse population, leading to increased fitness (Whiteley et al., 2015). The genomic erosion apparent at the start of the SMR recovery program was severe, and continued inbreeding and genetic drift in the recently translocated populations have worsened this. Consequently, we lack a population for this species from which to source beneficial novel genetic diversity (e.g. wild‐type alleles that can mask the effects of recessive deleterious mutations). Therefore, for the SMR, genetic rescue attempts necessarily entail movement between the genetically depleted populations, with the aim of minimizing genetic drift, maximizing observed heterozygosity and halting the increase of realized load.

Several studies (Stewart et al., 2017; Weeks et al., 2011) have shown that a single genetic rescue event does not achieve the long‐term goal of increased genetic diversity, and that continued immigration of new alleles is necessary for the longevity of small populations. A recent study on Scandinavian wolves found that some relief (i.e. increased genetic diversity, decreased genetic load) was experienced with a single translocation event performed for the purpose of genetic rescue. However, ultimately in the absence of ongoing connectivity, inbreeding and the associated side effects quickly built up again (Smeds & Ellegren, 2023). Our simulations support this finding: with both population sizes and natural migration limited in the SMR, a single translocation event between existing populations would likely only dampen the effects of drift and inbreeding for a few generations.

Recent studies propose that translocations for genetic rescue should consider genetic load, rather than genetic diversity alone (Hansson et al., 2021; Kyriazis et al., 2022; Ralls et al., 2020). However, the simulations for donor selection presented in this paper did not detect a discernible difference between selecting against the genetic load, selecting for genetic diversity, or picking individuals at random. This is likely due to the high relatedness between individuals that originated from a common founder population, which would have reduced between‐individual variation and possible differences between rescue regimes. If these simulations are used to broadly guide donor selection by incorporating genetic diversity and load estimates, this finding suggests that a thorough cost–benefit analysis should be carried out to determine if additional genetic resources would be important to select appropriate donors for translocations in the SMR. Long ROHs, such as those observed in the genomes of the modern SMR, are recognized to be disproportionately likely to harbour deleterious variation (Sams & Boyko, 2019; Szpiech et al., 2013). As such, selection for increased diversity (i.e. against long ROH) may act in parallel to selection against a high realized load. This might also partly explain why our simulations did not detect discernible differences between selection regimes.

Highly inbred populations may adversely react to assisted gene flow due to a high genetic load (Adams et al., 2022), and new (or previously purged) detrimental variation is (re)introduced to a population (Dussex et al., 2021; Ochoa et al., 2022). We, therefore, recognize that planning and preparation for translocations to support genetic rescue would ideally benefit from the use of genome resequencing data generated from the donor and receiving island populations, allowing the identification of any unique and shared harmful alleles. Furthermore, in species that are less genetically depauperate than the SMR, and in species with more divergent populations, this genomic assessment may be particularly prudent. Ultimately, such assessments of empirical genetic data of potential donors, coupled with the implementation of forward‐modelling simulations of the potential future effect on genetic load, will improve our understanding of the long‐term implications of genetic rescue. This, in turn, will allow genomic‐based selection of candidate donor individuals, so as to increase the long‐term success of genetic rescue attempts.

Intraspecific genetic rescue may offer some genetic stability for the SMR, although it is currently not technologically possible to reintroduce the full variation that has been lost through the bottleneck. While interspecific genetic rescue from a closely related species could be a saving grace for the SMR, outbreeding depression is a major concern of such actions. Tallmon et al. (2004) reviewed cases of interspecific genetic rescue, and argued much of the success was likely due to high levels of inbreeding in laboratory species, experiencing an immediate benefit from any external genetic injection. Given the severity of inbreeding in the modern SMR populations, we speculate that this may also be true. However, novel deleterious mutations introduced in the heterozygous state may become expressed if inbreeding continues, as might be expected given their limited migration and finite population size. Pavlova et al. (2023) present a case of successful interspecific rescue through captive breeding trials between the Critically Endangered helmeted honeyeater *Lichenostomus melanops cassidix* and the yellow tufted honeyeater *Lichenostomus melanops gippslandicus*. A number of individual fitness indicators improved among admixed offspring. The Madagascar magpie‐robin *Copsychus albospecularis* is a potential candidate for interspecific genetic rescue for the SMR. Given its geographical proximity to Seychelles, it is likely to have adapted to a similar ecological environment. However, the divergence time between both species is markedly greater than in the case of the honeyeater (Sheldon et al., 2009), and hybridization between such distant lineages is generally considered maladaptive. Nevertheless, the introduction of genetic variation from the Madagascar magpie‐robin could provide some novel adaptive variation to the extant SMR and recover some ancestral variation.

Many factors need to be considered when planning conservation actions. A crucial question is whether the projected long‐term benefits of rescue justify the ecological and economic costs. Intraspecific rescue is projected to reduce genetic load in the SMR by ~10% compared to no intervention. But what are the benefits for the species' long‐term viability? And how do we balance this against the more immediate ecological and welfare concerns? Not only do the SMR have delicate social structures and behavioural patterns that might be disrupted in the process, but any handling of the birds during trapping and transit may cause them distress. Furthermore, given we anticipate the SMR will benefit principally from a sustained series of genetic rescue events, this requires stakeholders to guarantee long‐term commitment to any effort started. And should alternatively interspecific genetic rescue be considered, effectively resulting in the formation of new hybrid birds, then one realistic consideration is whether genetic swamping may occur, effectively driving the original SMR to extinction. Alternatively, even if hybrids are successfully created, there is no guarantee that they would perform critical ecosystem functions originally performed by the SMR. Furthermore, there is difficulty surrounding wildlife protection laws (Wayne & Shaffer, 2016). As with any conservation action, a full assessment of the risks involved will need to be evaluated.

In summary, while the demographic rescue of the SMR has substantially improved the outlook of this species, at this point genetic consideration must be taken into account to fully evaluate the risk of genomic erosion. The SMR will undoubtedly benefit from continued genetic monitoring of the existing and any future populations and will require the implementation of genetic management strategies into the ongoing recovery program to ensure the survival of the species.

## CONFLICT OF INTEREST STATEMENT

The authors have no conflicts of interest to declare.

## Supporting information


Data S1.

## Data Availability

Raw sequencing reads have been deposited at the NCBI Sequence Read Archive (SRA) and are available under BioProject PRJNA1121496.
