# Supplementary material for: When birds of a feather flock together: Severe genomic erosion and the implications for genetic rescue in an endangered island passerine
Source: Evol Appl. 2024 Jun 28;17(7):e13739. doi: 10.1111/eva.13739 (PMC11212007; doi:10.1111/eva.13739)
Supplement: Supplementary file 1 — Data S1. [file EVA-17-e13739-s001.pdf]

Supporting information for

When birds of a feather flock together: severe genomic erosion and the implications for genetic rescue in an endangered island passerine.

Emily L. Cavill, Hernán E. Morales, Xin Sun, Michael V. Westbury, Cock van Oosterhout, Wilna Accouche, Anna Zora, Melissa J Schulze, Nirmal Shah, Pierre-André Adam, M. de L. Brooke, Paul Sweet, Shyam Gopalakrishnan, M. Thomas P. Gilbert

<u>Contents</u>

Supplementary Tables

Supplementary Table S1: Documented specimen information and sequencing data information (including summary statistics, sexing and locality results) for the 37 historical SMR samples

Supplementary Figures

Supplementary Figure S1: Full admixture plot for the historical dataset up to k=5.
Supplementary Figure S2: Simulations demonstrating the effect of direct counts versus relative counts of genetic load.
Supplementary Figure S3: Distributions of life-history trait parameters (fecundity and mortality probabilities) used for the simulations presented in Figure 7.
Supplementary Figure S4: Simulations demonstrating that random donor selection performs as well as targeted donor selection for genetic rescue in the SMR.
Supplementary Figure S5: Simulations demonstrating that ancestral variation will not be recovered through intraspecific genetic rescue.

Supplementary Table S1. Documented and sequencing data information for the 37 historic SMR samples. The 37 successfully sequenced museum specimens were collected from populations from four granitic (native) and one coralline (introduced) islands, but five of these specimens had an unknown collection origin, including our oldest 'LaFresnaye' sample. The initial genetic population assignment of our museum samples allowed us to either confirm or reassign the sampling site of each specimen, or assign a locality where that information is not already available. The combination of the results of these analyses allowed us to confirm there is likely to have been a mislabelling with two of the historical samples between Aride and Marianne, collected on the same voyage. We therefore re-assigned populations to these samples going forward. The 'LaFresnaye' sample is the oldest contribution to our historical dataset, thus identifying the locality was of great interest. While neither the PCA nor the Admixture analyses alone gave a definitive result to its origin, when combined with the phylogenetic analysis we are now confident that the sample was collected from Fregate Island. We were able to determine sex through a coverage-based method using ANGSD and offer new information where it wasn't previously available, or confirm/correct documented information. Some samples did not reach coverage considered reliable for this sexing method and are not presented. The knowledge gained from the sequencing data was passed to the respective museums, and we value the genetic origin assignment of museum samples as a good example of how genetics can be used to 'complete' museum collections.

In this table, from left to right, we include **SAMPLE:** the sample name is that used throughout the study. **INSTITUTE:** *Loan Institutions are abbreviated to a 4-letter acronym: AMNH = American Museum of Natural History, New York; MZCU = Museum of Zoology, Cambridge University; HMCZ = Museum of Comparative Zoology, Harvard University; NHML = Natural History Museum, London; MNHN = Muséum d'Histoire Naturelle, Paris; SNMH = National Museum of Natural History, Smithsonian Institution; YPBM = The Peabody Museum of Natural History, Yale University. **MUSEUM NO**: the collection numbers associated with each specimen. **DOC DATE:** the documented date we have associated with each specimen. **DOC ISLAND and SEQ ISLAND:** the documented and confirmed or reassigned through our sequencing data. **While Praslin only has one sample so we cannot 'confirm or deny' the collection locality through our data, we know where this sample came from, the collector only collected one sample from Praslin on this date. **DOC SEX and SEQ SEX:** the documented sex associated with the specimen and the sexing results from our sequencing data. **TOTAL READS:** we also include the total reads generated for each sample. **ENDOG:** and the total endogenous content of the sample, calculated as percentage of total reads that map uniquely to the SMR genome. **COV:** the final coverage for each sample. >78% of the historic samples met the 4X threshold used for many of our analyses.

SRA ID	INSTITUTE*	MUSEUM NO	DOC DATE	DOC ISLAND	SEQ ISLAND	DOC SEX	SEQ SEX	TOTAL READS	ENDOG	COV
hist01	AMNH	SKIN 580154	1905	Aride	Aride	male	male	342,227,032	44.23%	5.23
hist02	AMNH	SKIN 580156	1904	Marianne	Marianne	male	male	244,035,581	19.85%	4.53
hist03	YPBM	ORN 040909	1958	Fregate	Fregate	female	female	260,168,868	58.44%	9.11
hist04	AMNH	SKIN 580158	1904	Marianne	Marianne	male	male	166,320,727	40.30%	3.56

hist05	AMNH	SKIN 580159	1904	Marianne	Marianne	female	female	362,069,454	43.71%	8.54
hist06	AMNH	SKIN 580161	1904	Alphonse	Alphonse	female	female	299,941,434	48.91%	9.23
hist07	AMNH	SKIN 580162	1904	Alphonse	Alphonse	female	male	197,750,998	44.75%	5.29
hist08	AMNH	SKIN 580163	unknown	unknown	Marianne	unknown	female	97,320,372	47.01%	9.07
hist09	MNHN	MNHN-ZO-MO-1878-563	pre-1879	Aride	Aride	male	female	271,488,059	35.9%	5.14
hist10	MNHN	MNHN-ZO-MO-1878-575	pre-1879	Aride	Marianne	female	male	290,865,245	36.9%	5.71
hist11	YPBM	ORN 040908	1958	Fregate	Fregate	female	-	297,318,637	12.49%	2.03
hist12	MNHN	MNHN-ZO-MO-1878-580	pre-1879	Aride	Aride	female	female	193,930,086	39.3%	6.49
hist13	MNHN	MNHN-ZO-MO-1878-587	pre-1879	Marianne	Aride	male	male	270,454,754	50.5%	6.15
hist14	AMNH	SKIN 580155	1904	Aride	Aride	male	female	250,521,622	35.49%	5.24
hist15	AMNH	SKIN 580160	1904	Marianne	Marianne	female	female	386,515,886	55.21%	8.06
hist16	HMCZ	Ornithology 76293	pre-1861	unknown	Fregate	unknown	male	382,252,003	43.74%	11.08
hist17	LNHM	1868.6.16.8	1867	Marianne	Marianne	male	male	418,847,337	50.38%	9.53
hist18	LNHM	1888.4.20.96	1879	Marianne	Marianne	-	female	210,793,931	39.98%	4.62
hist19	LNHM	1895.5.1.909	pre-1895	Aride	Aride	male	male	247,996,934	53.62%	7.3
hist20	LNHM	1895.5.1.910	pre-1895	unknown	Marianne	female	female	189,048,592	50.90%	5.05
hist21	LNHM	1895.5.1.911	pre-1895	unknown	Marianne	-	female	240,566,111	40.36%	5.00
hist22	LNHM	1909.4.6.30	pre-1909	unknown	Marianne	-	male	201,450,869	54.40%	6.08
hist23	LNHM	1909.4.6.31	pre-1909	unknown	Marianne	-	female	196,027,704	60.49%	7.11
hist24	LNHM	1927.12.18.383	1888	Marianne	Marianne	male	male	230,852,236	52.13%	6.14
hist25	LNHM	1927.12.18.384	1888	Marianne	Marianne	female	female	188,259,165	52.31%	5.2
hist26	LNHM	1927.12.18.385	1888	Marianne	Marianne	male	male	188,448,978	46.87%	4.52
hist27	LNHM	1946.75.18a	1940	Fregate	Fregate	female	female	334,444,097	51.03%	7.97
hist28	LNHM	1955.63.4	1955	Fregate	Fregate	female	female	185,612,156	59.08%	6.26
hist29	LNHM	1996.51.1	1991	Fregate	Fregate	male	male	211,554,221	40.24%	5.38
hist30	LNHM	1946.75.16a	1940	Fregate	Fregate	female	-	87,780,683	23.82%	1.23
hist31	SNMH	119759	1890	Marianne	Marianne	female	-	10,599,191	31.11%	0.17

hist32	SNMH	128666	1892	Alphonse	Alphonse	male	male	254,465,448	26.23%	4.15
hist33	MZCU	27/Tur/17/g/2	1867	Praslin**	-	male	male	150,646,231	32.52%	4.05
hist34	MZCU	27/Tur/17/g/3	1867	Marianne	Marianne	female	-	39,244,697	52.78%	1.49
hist35	MZCU	27/Tur/17/g/4	1867	Marianne	Marianne	male	-	55,254,491	53.62%	2.08
hist36	MZCU	27/Tur/17/g/5	1867	Marianne	Marianne	female	-	49,463,953	56.76%	1.73
hist37	MZCU	27/Tur/17/g/6	1867	Marianne	Marianne	male	-	39,249,407	53.86%	1.57

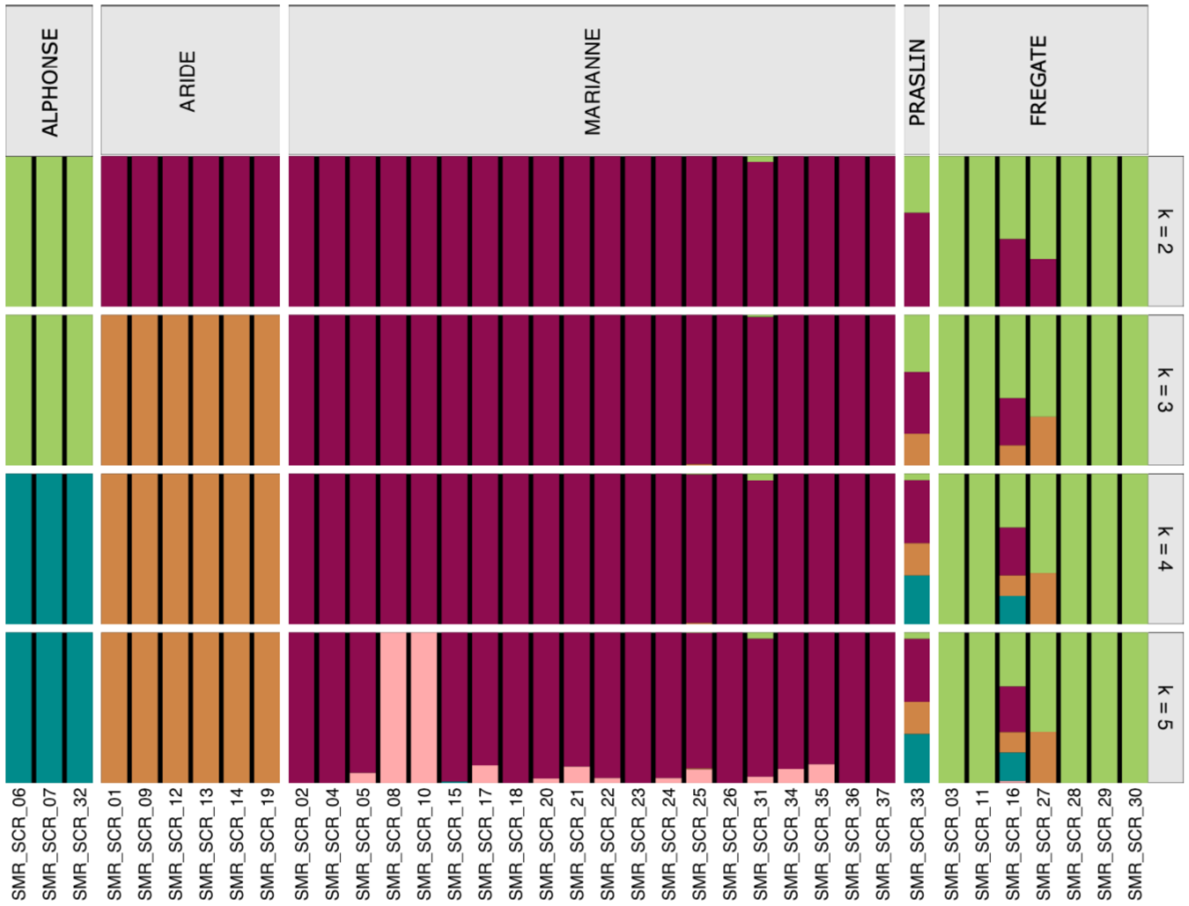

Supplementary Figure S1. Full admixture plot for the historical dataset. Admixture plot assuming 2 to 5 ancestry components where each bar represents the ancestry proportions per sample. k=3 separates the Aride population, k=5 supports no further insight for genetic differentiation between populations, only within the Marianne Island population. Sample 'SMR_SCR_16' (hist16) is the oldest collected sample of the dataset: 'pre-1861'.

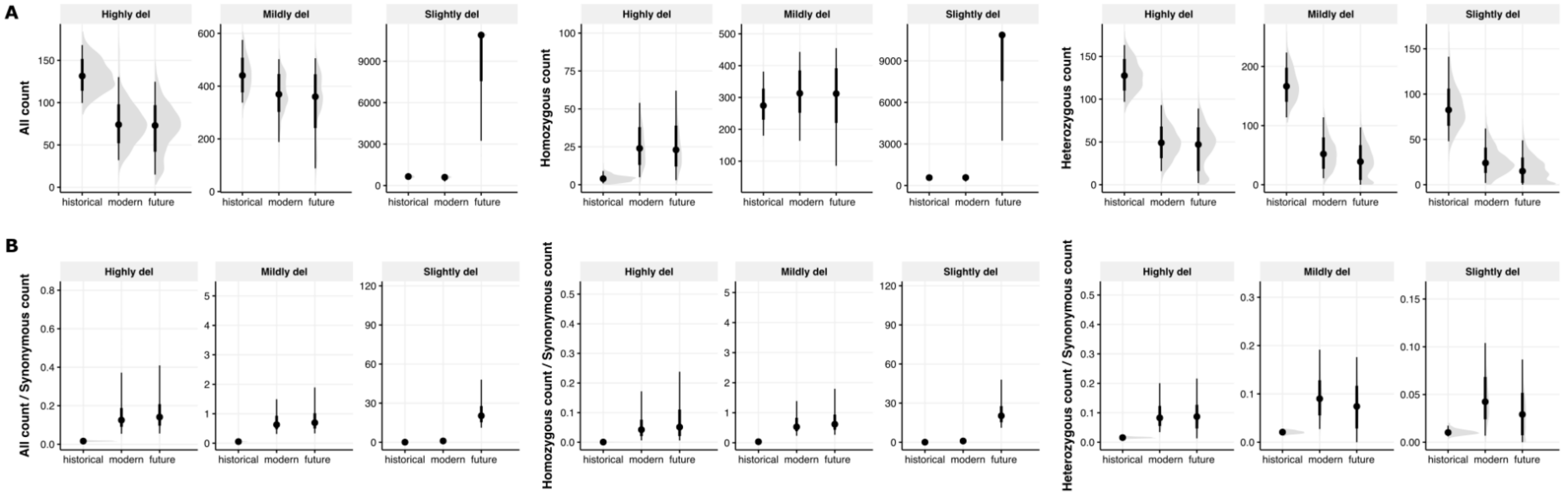

Supplementary Figure S2. Effect of direct counts versus relative counts of genetic load. We simulated the effect of **A)** counting mutations versus **B)** considering relative counts /normalised over synonymous. An ancestral population has high masked load and low realized load, as they go through a bottleneck, lots of the rare variation is simply lost (by drift) reducing the masked load. Another portion of it is converted from masked load to realised load. Of the converted load some is "seen" by selection and removed (purging; mostly highly deleterious variation) whilst other escaped selection and accumulated (mostly slightly/mildly deleterious variation). This is why the expectation is for both purging and accumulation. The bottleneck population will have less total genetic load, because it has less masked load, but it will have higher realised load (i.e., is on average less fit than the ancestral). This we cannot estimate from empirical data because we cannot fully estimate masked and realised load, thus with the empirical data we count alleles and distinguish LoF, missense and heterozygous or homozygous load to approximate these dynamics. If we were to simply count alleles directly, we would see what is described above. However, due to the characteristics of historical sequencing data, we do relative counts to synonymous mutations. So, while the ancestral population may have more LoF alleles, this is alongside more synonymous alleles. While the contemporary populations may have fewer LoFs (due to purging), there are also fewer synonymous alleles. Thus, we observe a higher relative count. We are therefore underpowered to detect purging and cannot really conclude that there is only load accumulation. However, we know from genetic theory and simulations above that we can assume that there has been drift and purging, but also accumulation of load, and therefore that the modern populations are most likely, on average, less fit than the ancestral populations

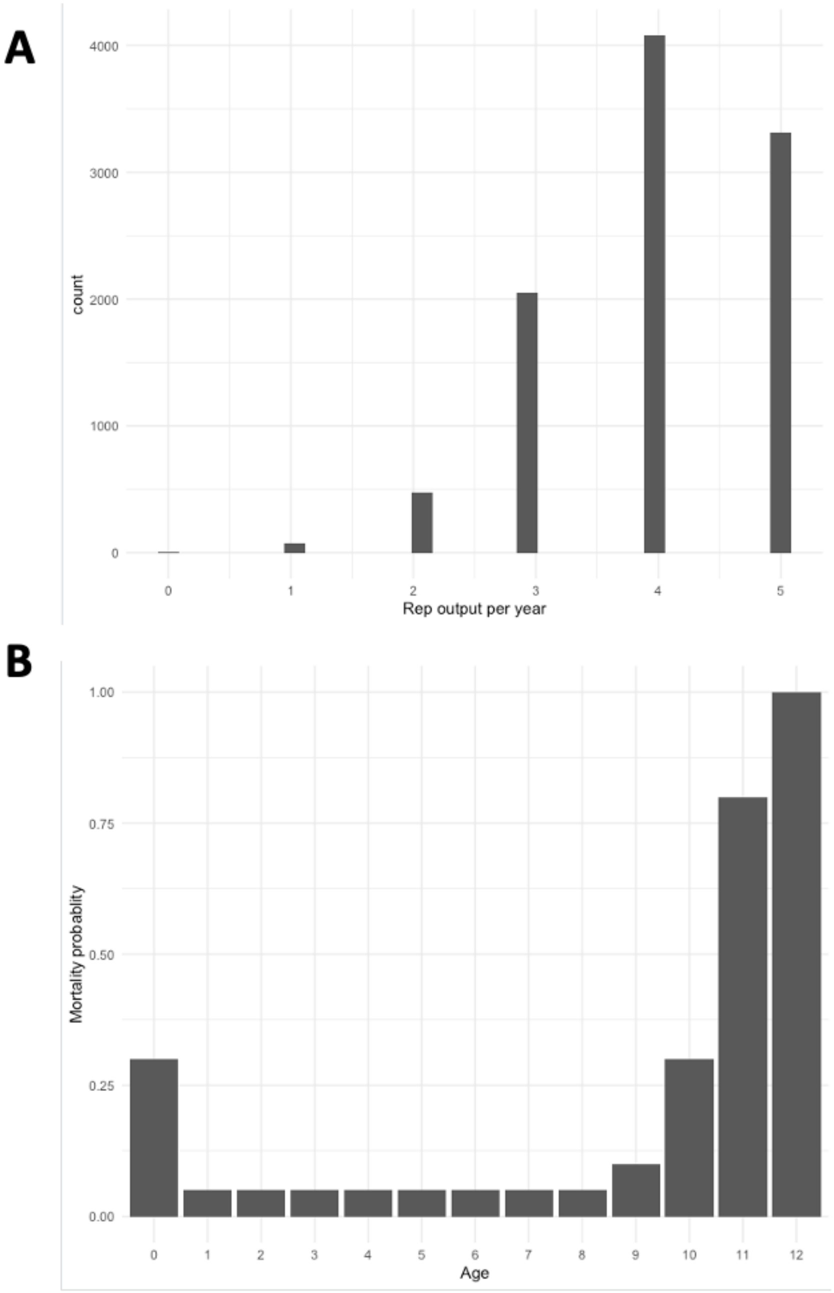

Supplementary Figure S3. Distributions of life-history trait parameters used in the simulations. A) Fecundity: reproductive output probability per pair, per year. B) Mortality probabilities.

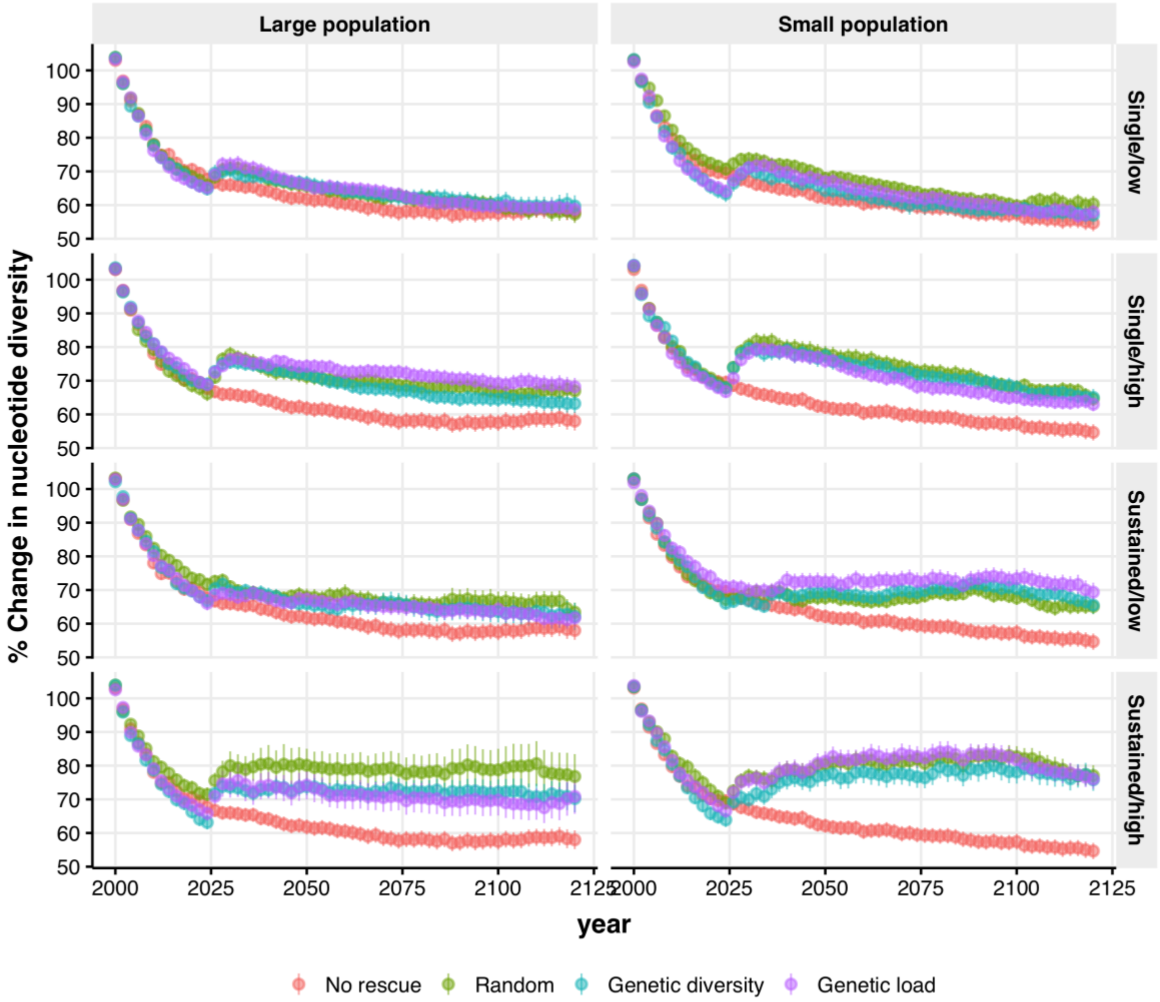

Supplementary Figure S4. Random donor selection performs as well as targeted donor selection. The "no rescue" regime is almost exclusively worse than any rescue regime. For targeted and random donor selection, the immediate effects (within a few generations) are substantial for nucleotide diversity, but they taper off and nearly disappear over time. In contrast, the reduction in realised load is less sharp but the effect is lasting. However, there is minimal difference between the “random”, “genetic load” and “genetic diversity” donor selection regimes. Interestingly, in the “sustained/high” scenario for the large population, random selection actually out-performs targeted selection.

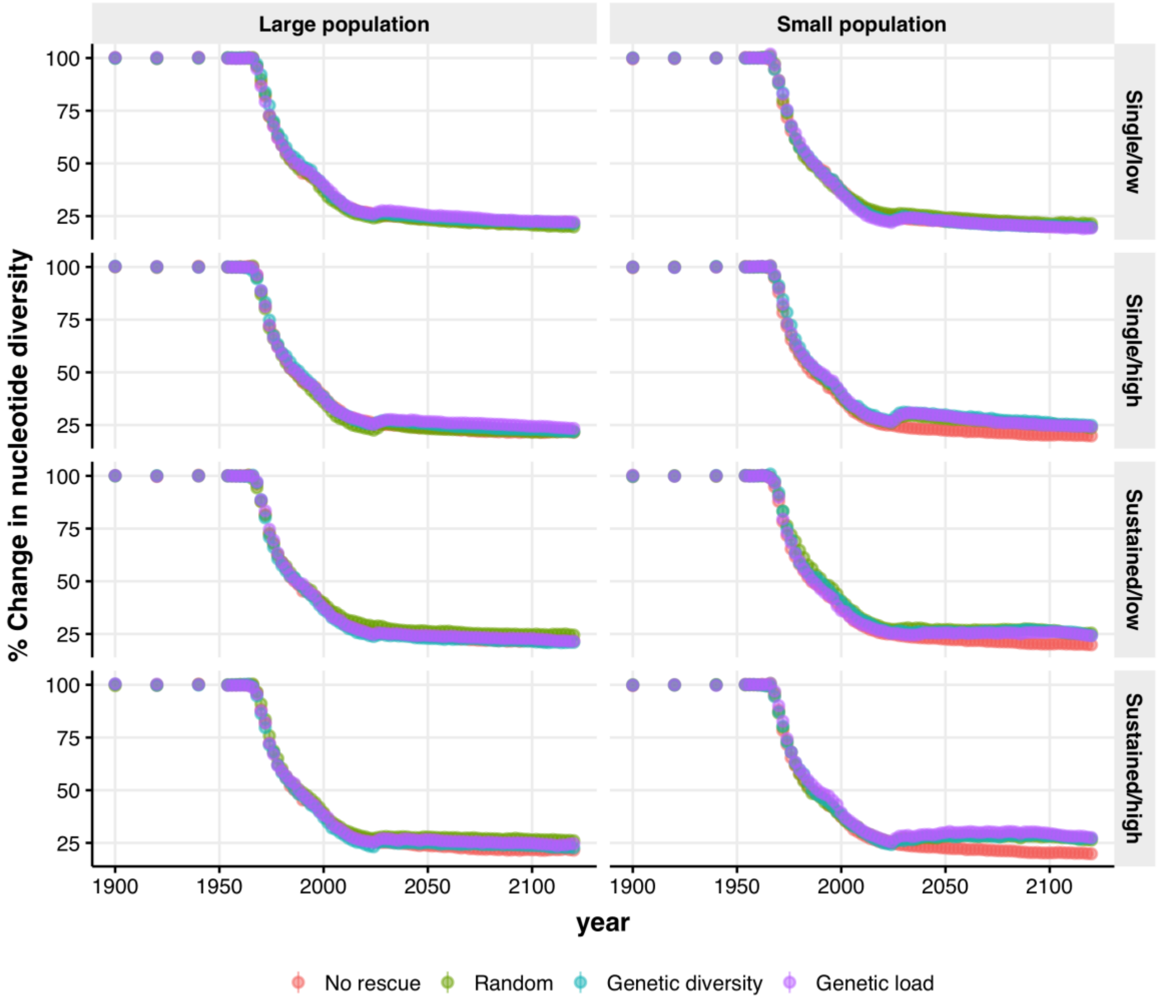

Supplementary Figure S5. Ancestral variation will not be recovered through intraspecific genetic rescue. Simulations show that intraspecific genetic rescue between the existing SMR populations may be enough to manage ongoing diversity loss, but this action alone will not be enough to recover the level which existed before the bottleneck.
